# Supplementary material for: Novel HSP90-PI3K Dual Inhibitor Suppresses Melanoma Cell Proliferation by Interfering with HSP90-EGFR Interaction and Downstream Signaling Pathways
Source: Int J Mol Sci. 2020 Mar 7;21(5):1845. doi: 10.3390/ijms21051845 (PMC7084941; doi:10.3390/ijms21051845)
Supplement: Supplementary file 1 [file ijms-21-01845-s001.pdf]

## Supplementary Material

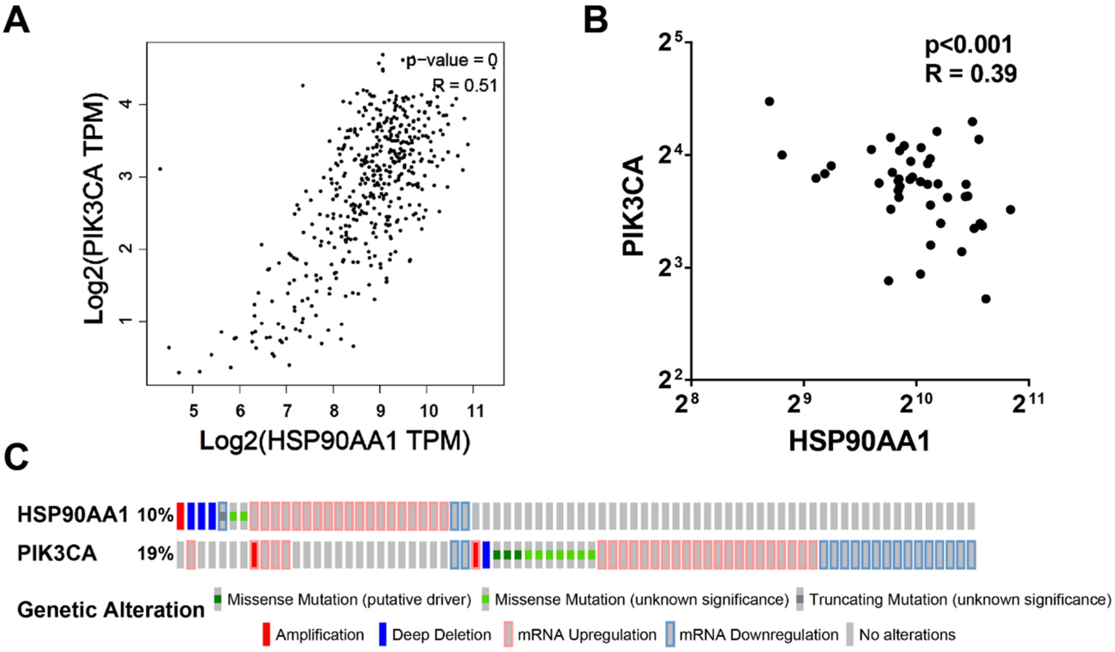


**Figure S1.** (A) The correlation of expression profiles on PIK3CA and HSP90AA1 in melanoma cohort of TCGA database. (B) The correlation of expression profiles on PIK3CA and HSP90AA1 in normal human skin tissues in TCGA and GTEx databse. (C) The mutations of HSP90AA1 and PIK3CA on melanoma patients in TCGA databse.

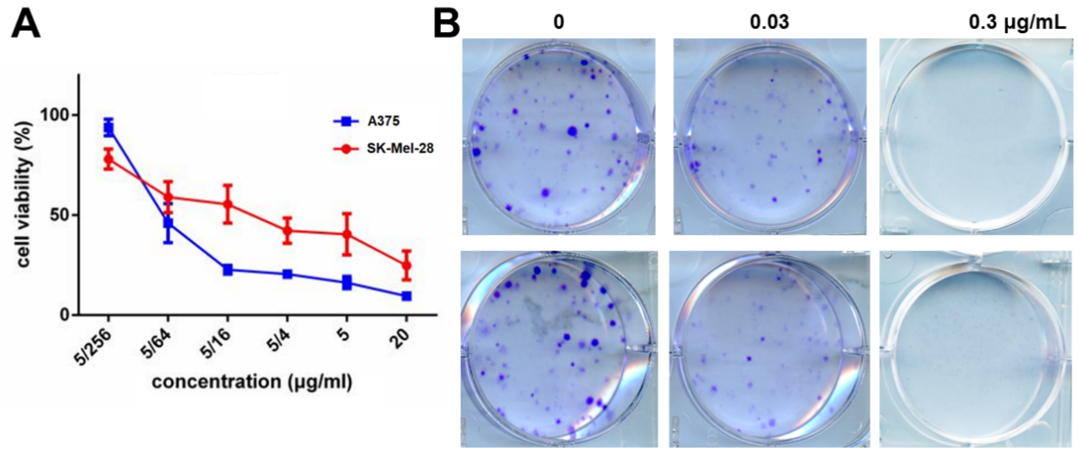


**Figure S2.** (A) The inhibition curves of cell viabilities with DHP1808 incubation, (B) The replicated results of colony formation assays, A375 cells were incubated with various concentrations (0.03 or 0.3μg/ml) of DHP1808 for 5 days.

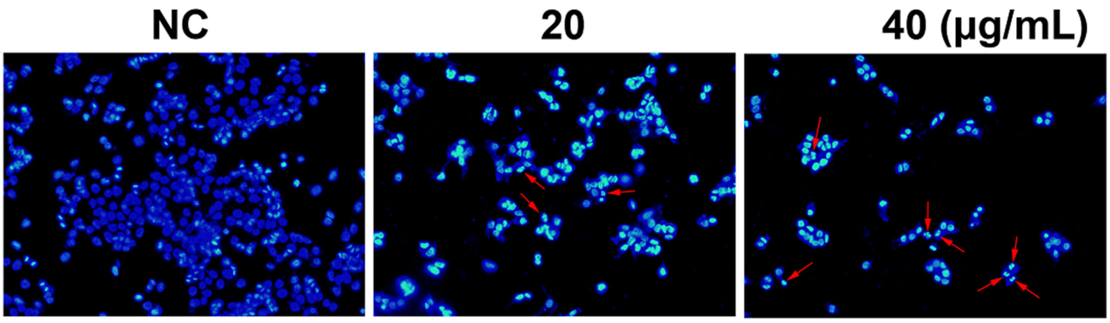


**Figure S3**. A375 cells were incubated with various concentrations (0, 20 or 40μg/ml) of DHP1808 for 24 h. The cellular morphology was observed under fluorescent microscopy after Hoechst 33258 staining.

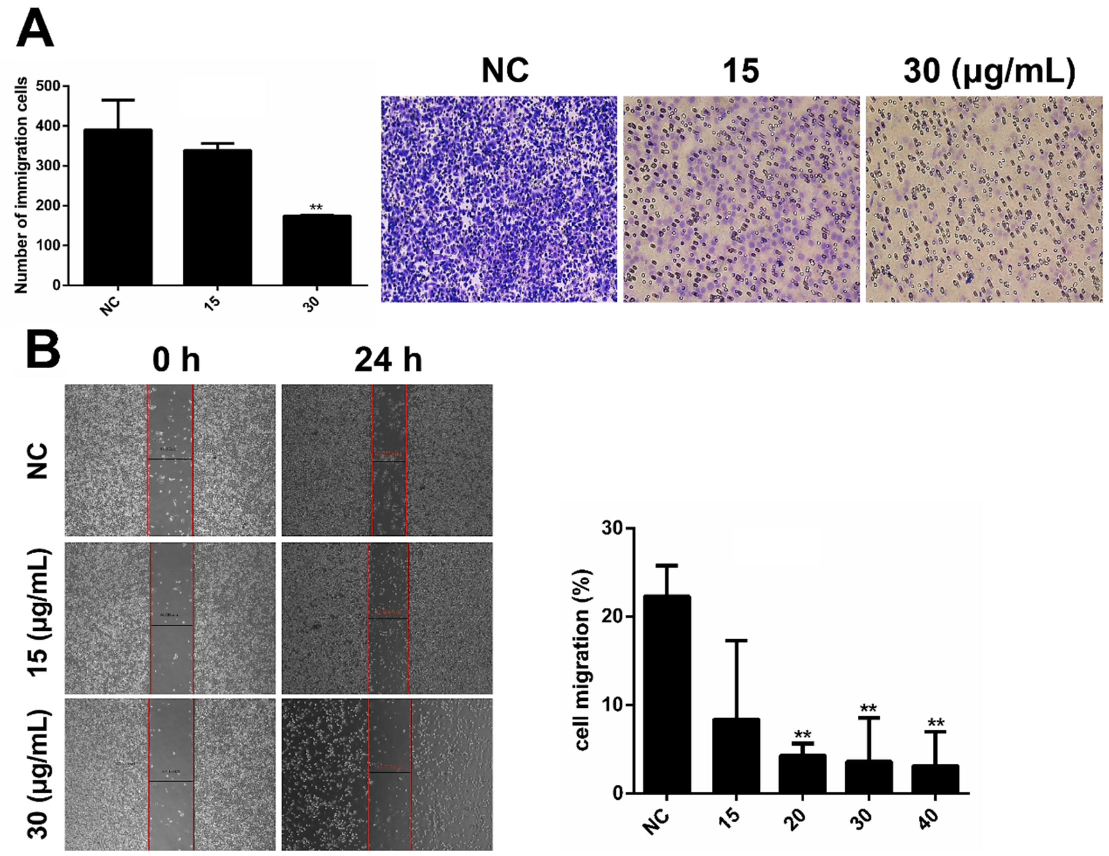


**Figure S4.** A. A375 cells were incubated with various concentrations (0, 15 or 30μg/ml) of DHP1808 for 24 h. The changes of cells invasion by transwell assay; B. A375 cells were incubated with various concentrations (0, 15 or 30μg/ml) of DHP1808 for 24 h. The changes of cells migration by wound healing assay;

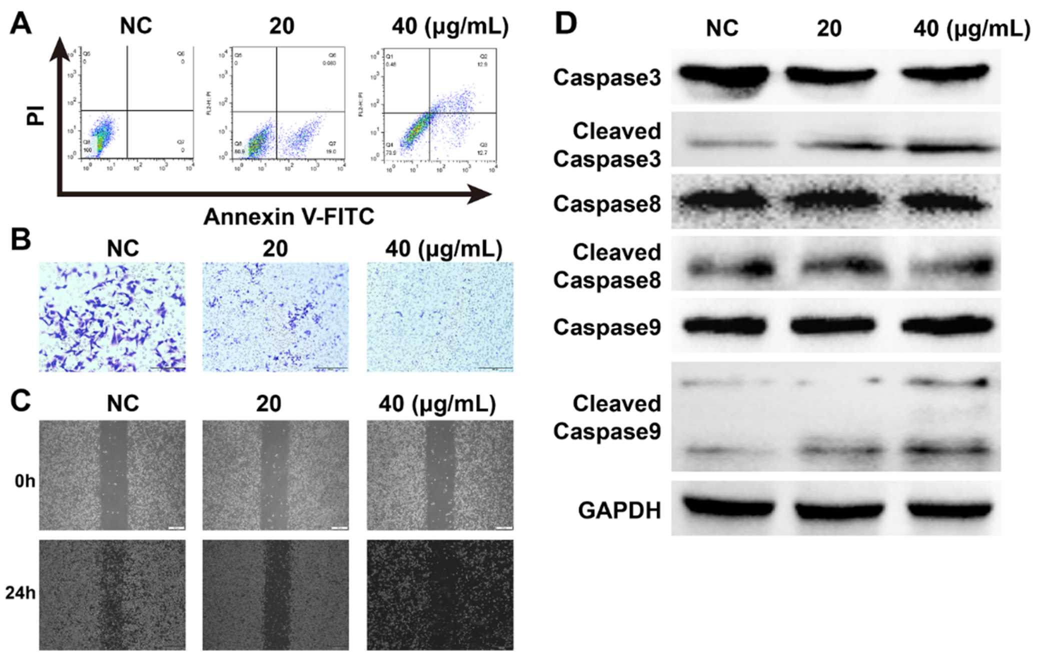


**Figure S5.** The results of Annexin V/PI staining (A), cell migration and invasion assays (B and C), caspase-3, caspase-8, caspase-9 immunoblot (D) with various concentrations (20 or 40μg/ml) of DHP1808 in SK-Mel-28 cells.

**Table S1**. Kinase Selectivity of DHP1808 against a panel of 97 kinases [a]

| Compound Name | DiscoveRx Gene Symbol | Percent Control (%) | Compound Concentration (nM) |
|---|---|---|---|
| DHP1808 | Abl | 86 | 1000 |
| DHP1808 | Abl(T315I) | 95 | 1000 |
| DHP1808 | ALK | 83 | 1000 |
| DHP1808 | ARK5 | 120 | 1000 |
| DHP1808 | Aurora-A | 97 | 1000 |
| DHP1808 | Axl | 121 | 1000 |
| DHP1808 | Blk | 113 | 1000 |
| DHP1808 | Bmx | 118 | 1000 |
| DHP1808 | BRK | 93 | 1000 |
| DHP1808 | B-Raf | 94 | 1000 |
| DHP1808 | B-Raf(V599E) | 103 | 1000 |
| DHP1808 | CDK1/cyclinB | 108 | 1000 |
| DHP1808 | CDK2/cyclinA | 112 | 1000 |
| DHP1808 | CDK5/p35 | 115 | 1000 |
| DHP1808 | CHK1 | 109 | 1000 |
| DHP1808 | CHK2 | 88 | 1000 |
| DHP1808 | CK1γ1 | 103 | 1000 |
| DHP1808 | CK1γ2 | 114 | 1000 |
| DHP1808 | CK1γ3 | 88 | 1000 |
| DHP1808 | cKit | 89 | 1000 |

| | | | |
|---|---|---|---|
| DHP1808 | cKit(D816H) | 92 | 1000 |
| DHP1808 | cKit(V560G) | 124 | 1000 |
| DHP1808 | CSK | 91 | 1000 |
| DHP1808 | c-RAF | 95 | 1000 |
| DHP1808 | cSRC | 120 | 1000 |
| DHP1808 | DAPK1 | 121 | 1000 |
| DHP1808 | DCAMKL3 | 85 | 1000 |
| DHP1808 | DDR2 | 96 | 1000 |
| DHP1808 | DYRK2 | 106 | 1000 |
| DHP1808 | EGFR | 105 | 1000 |
| DHP1808 | EGFR(L858R) | 114 | 1000 |
| DHP1808 | EGFR(L861Q) | 105 | 1000 |
| DHP1808 | EGFR(T790M) | 115 | 1000 |
| DHP1808 | EGFR(T790M,L858R) | 99 | 1000 |
| DHP1808 | EphA2 | 108 | 1000 |
| DHP1808 | EphA7 | 84 | 1000 |
| DHP1808 | EphB4 | 100 | 1000 |
| DHP1808 | ErbB2 | 91 | 1000 |
| DHP1808 | ErbB4 | 107 | 1000 |
| DHP1808 | FAK | 115 | 1000 |
| DHP1808 | Fer | 92 | 1000 |
| DHP1808 | Fes | 120 | 1000 |
| DHP1808 | FGFR1 | 95 | 1000 |
| DHP1808 | FGFR2 | 113 | 1000 |
| DHP1808 | FGFR3 | 95 | 1000 |
| DHP1808 | FGFR4 | 106 | 1000 |
| DHP1808 | Flt1 | 109 | 1000 |
| DHP1808 | Flt3(D835Y) | 95 | 1000 |
| DHP1808 | Flt3 | 105 | 1000 |
| DHP1808 | Flt4 | 114 | 1000 |
| DHP1808 | Fms | 110 | 1000 |
| DHP1808 | Hck | 95 | 1000 |
| DHP1808 | HIPK2 | 82 | 1000 |
| DHP1808 | HIPK3 | 96 | 1000 |
| DHP1808 | IGF-1R | 104 | 1000 |
| DHP1808 | IKKα | 121 | 1000 |
| DHP1808 | IKKε | 93 | 1000 |
| DHP1808 | KDR | 96 | 1000 |
| DHP1808 | LKB1 | 119 | 1000 |
| DHP1808 | MAPK2 | 122 | 1000 |
| DHP1808 | MEK1 | 100 | 1000 |
| DHP1808 | MELK | 91 | 1000 |
| DHP1808 | Mer | 90 | 1000 |
| DHP1808 | Met | 88 | 1000 |

| | | | |
|---|---|---|---|
| DHP1808 | MST3 | 122 | 1000 |
| DHP1808 | NIM1 | 105 | 1000 |
| DHP1808 | p70S6K | 91 | 1000 |
| DHP1808 | PAK1 | 118 | 1000 |
| DHP1808 | PAK4 | 118 | 1000 |
| DHP1808 | PDGFRα | 101 | 1000 |
| DHP1808 | PDGFRα(D842V) | 103 | 1000 |
| DHP1808 | PDK1 | 114 | 1000 |
| DHP1808 | Pim-1 | 93 | 1000 |
| DHP1808 | PKBα | 116 | 1000 |
| DHP1808 | PKCα | 90 | 1000 |
| DHP1808 | PKCε | 120 | 1000 |
| DHP1808 | PKCη | 102 | 1000 |
| DHP1808 | PKCι | 117 | 1000 |
| DHP1808 | PKCμ | 115 | 1000 |
| DHP1808 | PKCθ | 103 | 1000 |
| DHP1808 | PKD2 | 100 | 1000 |
| DHP1808 | Ret | 108 | 1000 |
| DHP1808 | ROCK-I | 98 | 1000 |
| DHP1808 | Ron | 85 | 1000 |
| DHP1808 | Ros | 99 | 1000 |
| DHP1808 | Snk | 84 | 1000 |
| DHP1808 | TAK1 | 110 | 1000 |
| DHP1808 | Tie2 | 121 | 1000 |
| DHP1808 | TrkA | 122 | 1000 |
| DHP1808 | TrkC | 106 | 1000 |
| DHP1808 | Wee1 | 115 | 1000 |
| DHP1808 | Yes | 94 | 1000 |
| DHP1808 | ZAP-70 | 82 | 1000 |
| DHP1808 | ZIPK | 104 | 1000 |
| DHP1808 | ATM | 85 | 1000 |
| DHP1808 | ATR/ATRIP | 87 | 1000 |
| DHP1808 | DNA-PK | 86 | 1000 |

[a] Measurements were performed at a concentration of 1 μM of the inhibitor in duplicate. The % control means active kinase percentage in the well. The affinity was defined with respect to a DMSO control.

**Experimental Section:**

**Colony formation assay**

For the colony formation assay, a total of 300 cells were seeded in 6-well plates in triplicate and maintained in the complete medium for 7-12 days. The natural colonies were washed with PBS and fixed with 4% paraformaldehyde for 30 min at room temperature. The colonies were then stained with Crystal Violet Staining Solution for 10 min, washed with water and air-dried. The total number of colonies with more than 50 cells was counted under fluorescence microscopy.

**Transwell assay**

For the invasion assay, we used Transwell chambers (8-μmpore size, Corning). Specifically, $1 \times 10^5$ of A375 cells were suspended in serum-free medium, and then loaded into the Matrigel precoated top-chambers (BD Bioscience, San Jose, CA, USA) and incubated with DMEM culture in the bottom chamber. Crystal violet staining was used to measure the invasiveness of A375 cells. Cell counts were performed in triplicate under a microscope.

**Wound healing assay**

Cell migration assay was performed using the agarose wound healing method. In brief, A375 cells were seeded in 6-well plate with DMEM for $1 \times 10^5$ cells per well and was further grown overnight. Three lines were cut in each well using a 200 ml pipette tip. Cells were then washed with PBS and serum-free DMEM including various concentrations of drugs or DMSO were added. Evaluation of the cell migration was determined under a light microscope between 0 h and 24 h of treatment.
